# Spectroscopic and Molecular Docking Investigation on the Interaction of Cumin Components with Plasma Protein: Assessment of the Comparative Interactions of Aldehyde and Alcohol with Human Serum Albumin

**DOI:** 10.3390/ijms23084078

**Published:** 2022-04-07

**Authors:** Mohd Sajid Ali, Md Tabish Rehman, Hamad Al-Lohedan, Mohamed Fahad AlAjmi

**Affiliations:** 1 Department of Chemistry, King Saud University, P.O. Box 2455, Riyadh 11451, Saudi Arabia; hlohedan@ksu.edu.sa; 2 Department of Pharmacognosy, College of Pharmacy, King Saud University, P.O. Box 2455, Riyadh 11451, Saudi Arabia; mrehman@ksu.edu.sa (M.T.R.); malajmii@ksu.edu.sa (M.F.A.)

**Keywords:** albumin, cumin components, cuminol, fluorescence, molecular docking, aldehyde and alcohol

## Abstract

The interaction of the important plasma protein, human serum albumin (HSA), with two monoterpenes found in cumin oil, i.e., cuminaldehyde (4-isopropylbenzaldehyde) and cuminol (4-isopropylbenzyl alcohol), was studied in this paper. Both experimental and computational methods were utilized to understand the mechanism of binding. The UV absorption profile of HSA changes in the presence of both cuminaldehyde and cuminol, due to the interaction between HSA with both monoterpenes. The intrinsic fluorescence intensity of HSA was also quenched on the sequential addition of both ligands, due to change in the microenvironment of the fluorophore present in the former. Quenching of HSA by cuminaldehyde was much higher in comparison to that in the presence of cuminol. Fluorescence quenching data were analyzed using modified Stern-Volmer and Lineweaver-Burk methods, which suggested that the binding mechanism was of a static type for both ligands. In both cases, the binding was favored by the domination of hydrophobic as well as hydrogen bonding/Van der Waals forces. Both ligands partially unfolded the secondary structure of HSA, although the effect of cuminaldehyde was more pronounced, as compared to cuminol. The preferred binding site of cuminaldehyde and cuminol inside HSA was also the same; namely, drug binding site 1, located in subdomain IIA. The study showed that cuminaldehyde binds strongly with albumin as compared to its alcohol counterpart, which is due to the more hydrophobic nature of the former.

## 1. Introduction

Due to their non-toxic and therapeutic properties, compounds obtained from natural sources, like plant and animal materials, have found a special place in many fields, such as the pharmaceuticals, medical, and cosmetics industries, etc. [1,2,3]. Most of the products obtained from natural sources are biocompatible in nature and are not harmful because their origins are, generally, the substances which are consumed by mankind. Commonly used spices and food condiments, such as black seeds, cumin, fennel, peppers, cinnamon, ginger, garlic, etc., contain a large number of compounds which have tremendous medicinal values, and are very popular nowadays [4].

Cumin seeds are very common and essential ingredients in many cuisines, particularly in the South Asian and Middle Eastern regions. They are also important ingredients in some cheeses, for instance, Leyden cheese. They are also found in some traditional French breads. Oil obtained from cumin is recognized to have several medicinal properties. Cumin itself is used as a traditional medicine, mainly in the Ayurveda and Unani systems of medicine, which are popular in Southeast Asia [5,6]. Cumin and its oil are good household items, which can be used for several gastrointestinal ailments. 

The essential oil of cumin is composed of many important substances, like cuminaldehyde, eugenol, cuminol, p-cymene, safranal, etc. [7]. Being the principal component of the essential oil of cumin, cuminaldehyde has been paid special attention by researchers, and its medicinal properties have also been recently reviewed [7,8]. Surprisingly, cuminaldehyde was found to possess the following broad range of therapeutic properties: antidiabetic, anticancer, anti-neurodegenerative, anti-inflammatory, antibacterial and antifungal. The other important metabolite, cuminol, is also known to have decent beneficial effects as an antioxidant [9]. It has also been reported to increase the secretion of insulin [10]. It bound and inhibited Escherichia coli ATP synthase and cell growth [11]. It can repel mosquitoes [12] and is the starting material for the synthesis of 1-(4′-isopropylbenzyl)-1, 10-phenantrolinium bromide, having excellent antimalarial activity [13].

The intake of food, or any other substance through the mouth, is followed by its journey through the digestive system, where it is broken down in the process of digestion. This process involves the absorption of small metabolites in the small intestine, which then delivers the metabolites to the bloodstream, to be distributed all over the body through the circulatory system. Blood contains several binding proteins which are responsible for transporting various substances all over the body to carry out their biological functions [14]. These proteins bind with therapeutic molecules and carry them to various sites to accomplish their pharmacological purposes [15,16]. Human serum albumin (HSA) is the most abundant binding protein present in blood and is known to bind reversibly with a variety of substances, that include fatty acids, bilirubin, drugs and many other substances [17,18]. From previous studies it has been found that different substances have different binding affinities with serum albumins, depending on the structures of the former [19,20,21,22,23,24]. The extent of binding of a pharmaceutically important molecule with serum albumin decides its therapeutic action, because strong binding may increase the half-life of the former in the body and might lead to unwanted side effects, whereas weak binding may result in early excretion of the former from the body and consequent inefficiency. Thus, it is very important to understand the binding of serum albumin with a molecule of medicinal value, like cuminaldehyde and cuminol. The interaction of bovine serum albumin with cuminol was recently studied and it was found that the interaction was very weak [25]. Therefore, we have designed this study to see the comparative interaction of HSA with cuminaldehyde and cuminol, because these belong to aldehydes and alcohols from the same series. Moreover, working directly on HSA would also provide straightforward information on the interaction, rather than considering its bovine counterpart as a model protein. For accomplishing this goal, we have utilized experimental UV-visible, fluorescent and CD spectroscopies, together with computational molecular docking methods.

## 2. Results and Discussions

### 2.1. UV-Visible Absorption Measurements

**Figure 1:**
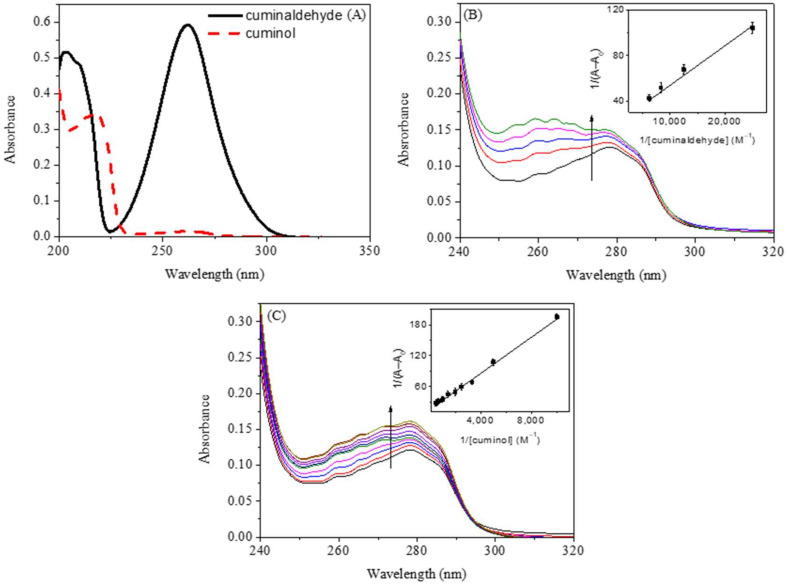
(**A**) UV-visible absorption spectra of cuminaldehyde and cuminol. The concentration of both substances was 40 µM. (**B**) Different UV-visible spectra of HSA in the absence and presence of cuminaldehyde (0, 40, 80, 120 and 160 µM) and (**C**) cuminol (0, 100, 200, 300, 500, 700, 1000 and 2000 µM). The concentration of HSA was 3.0 µM at 25 °C.

The UV-visible spectra of cuminaldehyde and cuminol, given in Figure 1A, show strong absorption of both compounds in the range of 200 nm to 220 nm. Cuminaldehyde also shows strong absorption near 270 nm, while cuminol has very weak absorbance in this range. The different UV-visible spectra of HSA in the absence and presence of cuminaldehyde and cuminol are given in Figure 1B,C, respectively. Proteins show an absorption band at around 280 nm, which is due to the presence of aromatic amino acid residues (tryptophan, tyrosine and phenylalanine) [19,20]. Change in UV absorption profile of a protein in the presence of a ligand, or small molecule, is ascribed to change in the microenvironment of these residues, due to their interactions with the ligand or small molecule [23,24,26]. It can be seen from the figures that both cuminaldehyde and cuminol have shown some impact on the UV-absorption profile of the protein. However, cuminol required high concentrations to significantly affect the UV-absorption profile of HSA, in comparison to cuminaldehyde, which was very effective at lower concentrations. This is an indication of the stronger interaction of cuminaldehyde with HSA to that of cuminol.

The association constant (Ka) between HSA and ligand (cuminaldehyde or cuminol in the present case) can be calculated using the Benesi-Hildebrand equation [27], which is given as:

(1) $$\frac{1}{A_{obs}-A_{0}}=\frac{1}{A_{c}-A_{0}}+\frac{1}{A_{c}-A_{0}}\times \frac{1}{K_{a}\left[ligand\right]}$$

where *A_obs_* is the observed absorbance of the solution containing different concentrations of the ligands at 280 nm, *A*_0_ and *A*_c_ are the absorbances of HSA and the complex at 280 nm, which have been used in further analysis. [28]. The association constant (*K*_a_) can be obtained from the linear regression of plot of 1/(*A − A*_0_) and 1/[*ligand*] (insets of Figure 1B,C). The analyzed values of *K*_a_ at 25 °C was found to be 7.5 (±0.5) × 10^3^ M^—1^ for cuminaldehyde and 9.2 (±0.6) × 10^2^ M^—1^ for cuminol. From the calculated values of association constant, it can easily be understood that cuminaldehyde interacted strongly with HSA, in comparison to the interaction of the latter with cuminol.

### 2.2. Intrinsic Fluorescence Measurements

Proteins which have the aromatic amino acids described above are known to exhibit intrinsic fluorescence properties, due to the fluorescent nature of these amino acids. When present together, tryptophan is the most fluorescent, while tyrosine has little fluorescence, and the contribution of phenylalanine is negligible, as compared to the others. HSA has one tryptophan residue, Trp 214, located inside subdomain IIA. Excitation at 295 nm gives the fluorescence emission of the tryptophan residue, which is free from that of tyrosine residues. Thus, we have selected this wavelength for studying the fluorescence quenching of HSA by cuminaldehyde and cuminol. One important point to discuss here is that cuminaldehyde has small absorption at 295 nm, while cuminol has no absorption at this wavelength (Figure 1A). Thus, the fluorescence data were corrected for inner filter effect in case of cuminaldehyde-HSA interaction only, using the Equation (S1) [29].

**Figure 2:**
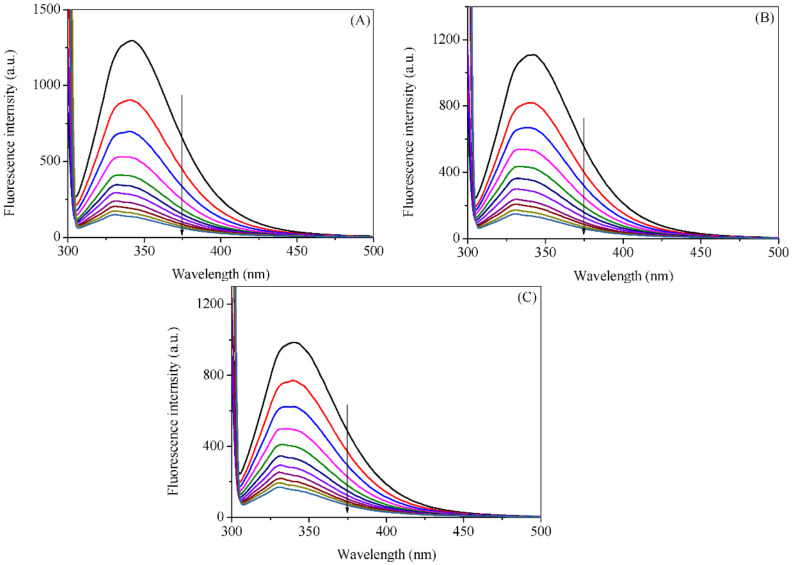
Fluorescence emission spectra of HSA in the absence and presence of cuminaldehyde at 25 °C (**A**), 35 °C (**B**), and 45 °C (**C**). λ_ex_ = 295 nm. The concentration of HSA was 3.0 µM, while concentration of cuminaldehyde varied from 0 to 400 µM with a regular increment of 40 µM.

**Figure 3:**
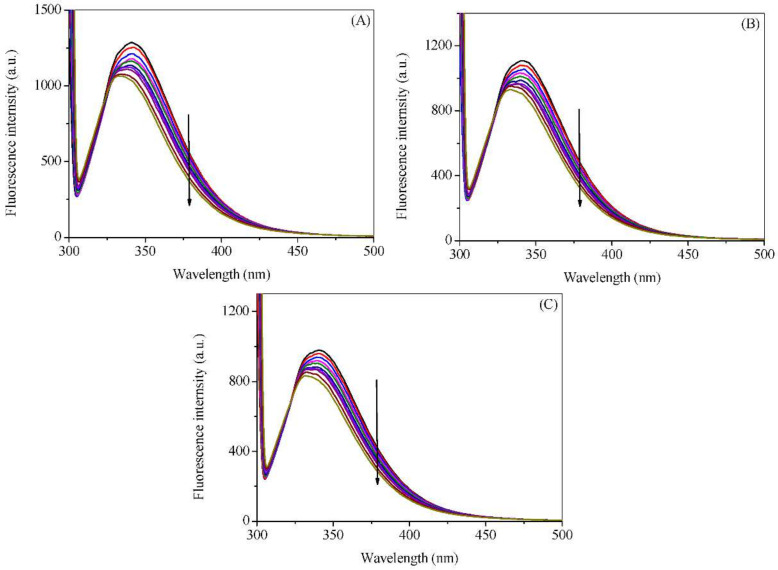
Fluorescence emission spectra of HSA in the absence and presence of cuminol at 25 °C (**A**), 35 °C (**B**), and 45 °C (**C**). λ_ex_ = 295 nm. The concentration of HSA was 3.0 µM, while concentration of cuminol was 0, 100, 200, 300, 400, 600, 800, 1000, 1500, 2000 µM.

**Figure 4:**
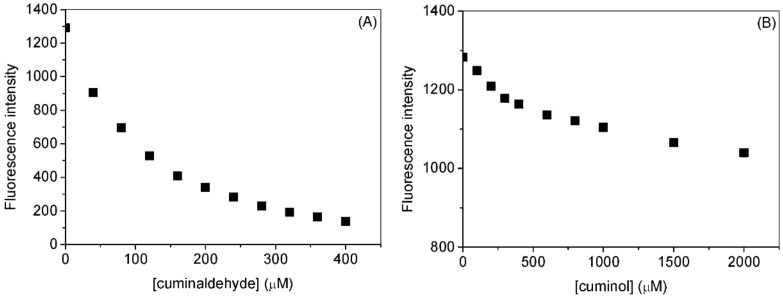
Effect of cuminaldehyde (**A**) and cuminol (**B**) on the fluorescence intensities of HSA at λ_ex_ = 295 nm and 25 °C. [HSA] = 3.0 µM.

The fluorescence spectra of HSA with various concentrations of cuminaldehyde and cuminol at various temperatures are given in Figure 2 and Figure 3, respectively. Addition of the cuminaldehyde, as well as cuminol, to the HSA solution causes the fluorescence of the latter to decrease. The decrease in the fluorescence of a fluorophore (quenching) on the addition of a small molecule or ligand is ascribed to change in the microenvironment of the former. The direct representation of the decrease in fluorescence on increasing the concentrations of cuminaldehyde and cuminol is given in Figure 4A,B, respectively. It is apparent from the figures that cuminaldehyde quenches the fluorescence of HSA very strongly as compared to cuminol, which has a very weak effect on quenching. Thus, it is anticipated that cuminaldehyde interacts strongly with HSA, while the interaction of cuminol is relatively weaker. 

Further, intrinsic fluorescence quenching was also quantized using the well-known Stern-Volmer equation, which utilizes the values of fluorescence intensities of fluorophore in the absence (*F*_0_) and presence of (*F*) quencher, along with the Stern-Volmer quenching constant (*K*_SV_) and concentration of quencher [*Q*]:

(2) $$\frac{F_{0}}{F}=1+K_{SV}\left[Q\right]$$

Stern-Volmer plots of HSA cuminaldehyde/cuminol interactions are given in supporting information (Figure S1 for cuminaldehyde and Figure S2 for cuminol). As can be seen from the figures, the plots are linear in the case of HSA–cuminaldehyde interactions. However, these plots are non-linear with negative deviation in the case of HSA–cuminol interaction, probably due to the presence of a secondary binding site to which the fluorophore is not uniformly accessible to the quencher [30,31,32]. Therefore, the data have been treated using a modified Stern-Volmer equation. Furthermore, for the sake of comparison between the interactions of these two ligands with HSA, we have also treated the HSA–cuminaldehyde fluorescence quenching data with the same equation, which is given as [22,33]:

(3) $$\frac{F_{0}}{F_{0}-F}=\frac{1}{f_{a}K_{Q}\left[Q\right]}+\frac{1}{f_{a}}$$

where *f*_a_ is the fraction of the initial fluorescence that was accessible to the quencher. Hence, the values of quenching constant (*K*_Q_) were graphically determined by Equation (3), and the plot of *F*_0_/(*F*_0_ − *F*) vs. 1/[*Q*] yielded f_a_^−1^ and (*f*_a_*K*_Q_)^−1^ as the intercept and slope, respectively.

**Figure 5:**
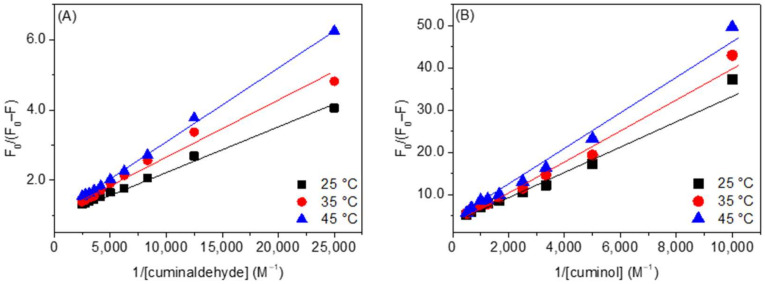
Modified Stern-Volmer plots of (**A**) HSA–cuminaldehyde and (**B**) HSA–cuminol systems at various temperatures.

The values of *K*_Q_, given in Table 1 and Table 2, were obtained through linear regression (Figure 5A for cuminaldehyde and Figure 5B for cuminol) of Equation (3). From the values given in Table 1 and Table 2, it is discernible that the *K*_Q_ in the case of cuminaldehyde–HSA interactions were much higher than the ones for the cuminol–HSA interaction. Further, these values are also in close agreement with the ones obtained from UV-visible spectroscopy.

It is very well recognized that quenching is classified either as static or dynamic, which can be distinguished by varying the temperature. A decrease in the temperature increases the static quenching constant while collisional encounters between fluorophore and quencher may increase at higher temperatures, giving rise to increase in quenching constants. Thus, the quenching mechanism can be understood by studying interaction at various temperatures. The decrease in the values of *K*_Q_ with an increase in temperature for both cases (Table 1 and Table 2) is an indication of the involvement of a static quenching mechanism in the interaction.

**Figure 6:**
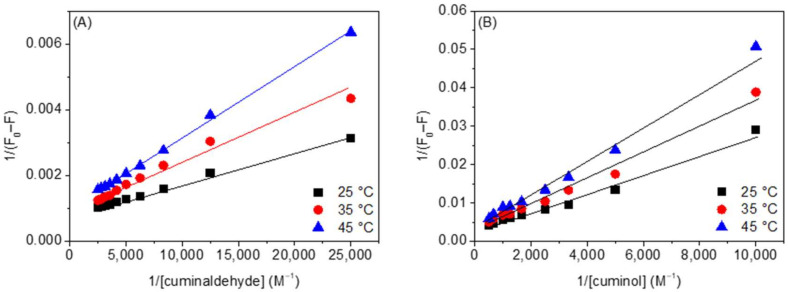
Lineweaver-Burk plots of (**A**) HSA-cuminaldehyde and (**B**) HSA-cuminol systems at various temperatures.

For more support of our supposition regarding the mechanism of interaction, we have further analyzed our data using the Lineweaver-Burk method, which can be given as [19,34,35,36]:

(4) $$\frac{1}{F_{0}-F}=\frac{1}{F_{0}K_{LB}\left[Q\right]}+\frac{1}{F_{0}}$$

where *K*_LB_ is the Lineweaver-Burk static quenching constant. The plots of 1/(*F*_0_ − *F*) vs. 1/[*Q*] (Figure 6A,B) were used to calculate *K_LB_*. The values of *K_LB_* and their regression coefficients are given in Table 1 (cuminaldehyde) and Table 2 (cuminol). The better linearity of the Lineweaver-Burk curves as compared to the Stern-Volmer curves (Figures S1 and S2) also suggests the participation of a static quenching mechanism in the interaction [34,35,36]. Additionally, the values of *K*_LB_ and *K*_Q_ are in excellent agreement with each other.

**Figure 7:**
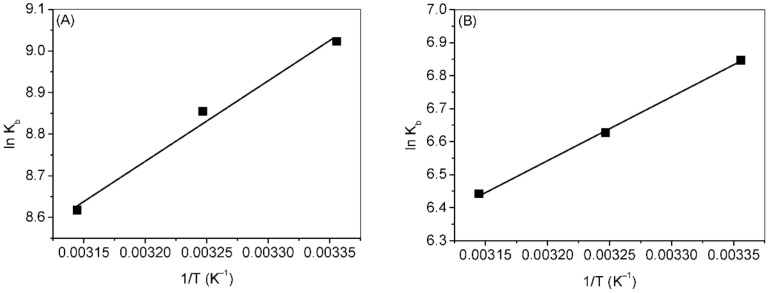
Van’t Hoff plot for the (**A**) HSA–cuminaldehyde and (**B**) HSA–cuminol interactions.

Evaluation of thermodynamic parameters is helpful in understanding the types of forces (hydrogen bonding, electrostatic, hydrophobic, Van der Waals forces, etc.). Thermodynamic parameters, such as enthalpy change (Δ*H*), entropy (Δ*S*) and free energy change (Δ*G*) can be calculated using the Van’t Hoff equations (Equations (S2) and (S3)). Figure 7A,B show the respective Van’t Hoff plots of cuminaldehyde–HSA and cuminol–HSA interactions and the values of the thermodynamic parameters are given in Table 3 and Table 4. The interaction of HSA with cuminaldehyde, as well as with cuminol, was spontaneous. However, the more negative values of Δ*G* in the case of cuminaldehyde suggested that the HSA–cuminaldehyde complex was more stable. The interaction between HSA and cuminaldehyde or cuminol was accompanied by negative values of Δ*H* and positive values of Δ*S* which suggest that interaction was favored by the dominance of both hydrophobic forces as well as hydrogen bonding, although the chances of the presence of any other type of binding forces cannot be ruled out [37,38].

It is evident from these results that cuminaldehyde interacts more strongly with HSA in comparison to cuminol. Tan and Siebert have reported that alcohols did not significantly bind to albumin, while aldehydes have shown stronger interactions [39]. The possible reason for this may be the more hydrophobic nature of the carbonyl group as compared to the alcohol group [40,41]. Additionally, the carbonyl group is more polar than the alcohol group. Thus, the stronger interaction of cuminaldehyde with HSA over that of cuminol with HSA is due to the greater hydrophobicity and polarity of the former.

### 2.3. Competitive Binding Site Experiments

**Figure 8:**
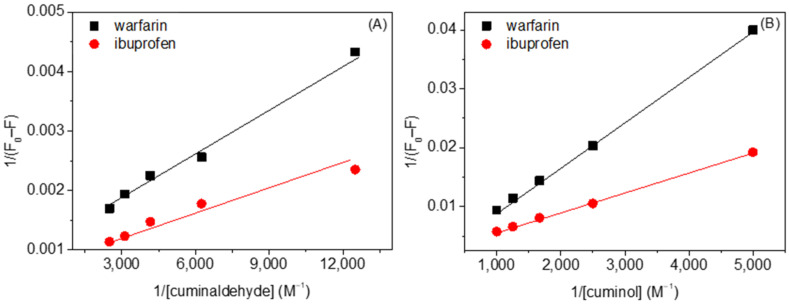
Lineweaver-Burk plots of (**A**) HSA–cuminaldehyde and (**B**) HSA–cuminol systems in presence of warfarin or ibuprofen.

HSA is a big molecule containing around 585 amino acids, and capable of binding molecules of different types. It has several binding sites, among which two sites, designated as drug site 1 (present in subdomain IIA) and drug site 2 (present in subdomain IIIA), are the most important [42,43]. There are several molecules which specifically bind at these sites, such as warfarin, which has affinity to bind at site 1, and ibuprofen, which binds strongly at site 2 [44]. Therefore, the binding site of molecules which bind at one of these two sites can be determined by competitive binding site experiments and by studying the binding of the former in the presence of the latter. Thus, competitive binding site experiments between ligands and site markers were carried out using fluorescence spectroscopy. The Lineweaver-Burk plots of interaction of HSA with cuminaldehyde and cuminol in the presence of warfarin and ibuprofen are given in Figure 8A,B, respectively, and the calculated values of the K_LB_ are given in Table 5. The values of K*_LB_* for HSA–cuminaldehyde/cuminol interactions decreased in the presence of warfarin, whereas there is very little, or no, effect in the presence of ibuprofen. Thus, the preferred binding site of both cuminaldehyde and cuminol inside HSA is drug site 1. These results are also in excellent agreement with results obtained from the computational method discussed in Section 2.5 of the manuscript.

### 2.4. Circular Dichroism Measurements

**Figure 9:**
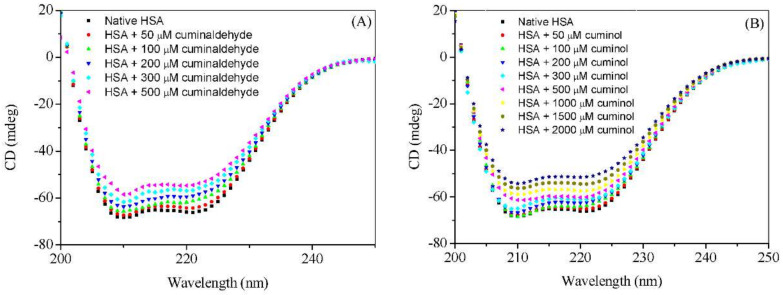
Far– UV CD spectra of HSA (3 µM) in absence and presence of (**A**) cuminaldehyde and (**B**) cuminol at 25 °C.

The comparative effect of cuminaldehyde and cuminol on the secondary structure of HSA was also studied using the far-UV CD method. HSA is a well-known α-helical protein with approximately 67% of α-helical contents [45], characterized by the presence of two negative peaks at 208 nm and 222 nm in the far-UV CD spectrum (Figure 8). The Far-UV CD spectra of interaction of HSA with cuminaldehyde and cuminol are given in Figure 9A,B, respectively. Both cuminaldehyde and cuminol influenced the secondary structure of HSA by decreasing the α-helicity of the protein; although only partially. At low concentrations the effect of cuminaldehyde was more pronounced, but this is probably because the level of binding of cuminaldehyde is higher than that of cuminol at those concentrations. At saturating concentrations for the two ligands, the effects were not that different.

The % values of α-helix are calculated using the standard method of Chen et al. [46], given in the supporting information (Equations (S4) and (S5)), and given in Table 6. The calculated values of % α-helix of HSA are in excellent agreement with the literature values. It is evident from the figures as well as values enlisted in Table 6 that both ligands partially affect the secondary structure of HSA, by decreasing the α-helical contents of the protein.

### 2.5. Molecular Docking

**Figure 10:**
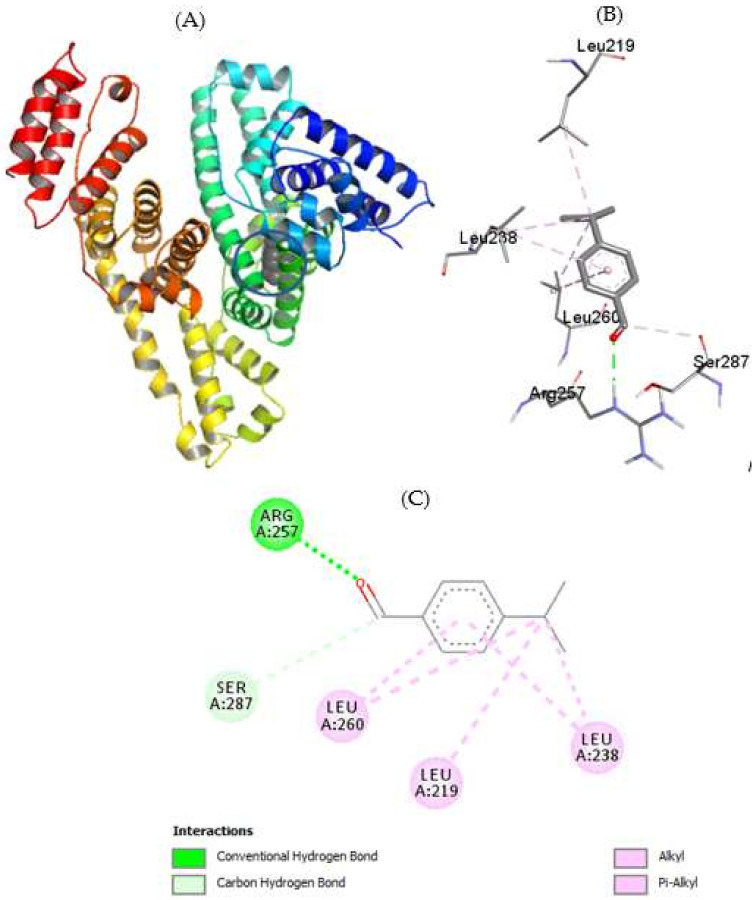
(**A**) Cluster analysis of interaction of cuminaldehyde with HSA (**B**) binding pocket amino acids (**C**) 2-D diagram showing the types of interactions involved.

**Figure 11:**
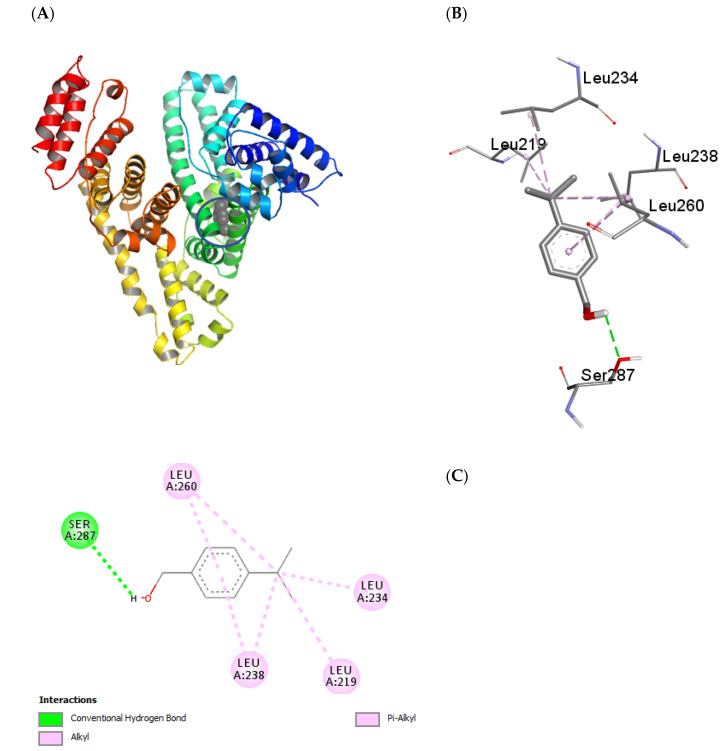
(**A**) Cluster analysis of interaction of cuminol with HSA (**B**) binding pocket amino acids (**C**) 2-D diagram showing the types of interactions involved.

The interaction of cuminaldehyde and cuminol with HSA was also seen using the widespread computational method known as molecular docking. HSA is a heart-shaped globular protein which possesses several binding sites, the most important of which have been designated as drug site 1 (located in Subdomain IIA) and drug site 2 (located in subdomain IIIA). Apart from these sites there are several other sites which are known to bind different fatty acids and small molecules [44]. Both cuminaldehyde and cuminol prefer to bind at drug site 1 (Figure 10A and Figure 11A). Similar to experimental observations, the docking energy in the case of cuminaldehyde was higher than that of cuminol. Since both ligands bind at the same site, most of the interacting amino acids were the same, which include, LEU219, LEU238, LEU260, and SER287, while cuminaldehyde also binds through ARG257 and cuminol binds through LEU234 with HSA (Figure 10B and Figure 11B). All leucine amino acid residues which took part in the interaction were bound through hydrophobic forces, while serine and arginine residues interacted through hydrogen bonding (Figure 10C and Figure 11C).

## 3. Materials and Methods

### 3.1. Materials

HSA (≥99%, A3782), cuminaldehyde (≥98.0%, 135,178) and cuminol or 4-Isopropylbenzyl alcohol (≥97.0%, 196,037) were purchased from Sigma, Plymouth Road Livonia, MI, USA. The experiments were carried out in a 20 mM Tris buffer of pH 7.4. The concentrated stock solutions of cuminaldehyde and cuminol were prepared in ethanol. The experiments using the techniques described below were performed in triplicate.

### 3.2. UV-Visible Spectroscopy

UV-visible measurements were performed with the Perkin-Elmer Lambda 65 UV-visible spectrophotometer (Waltham, MA, USA), equipped with a temperature controller water bath. Quartz cells of 1 cm were used for the spectral measurements.

### 3.3. Fluorescence Spectroscopy

Fluorescence experiments were performed on the Hitachi F 7000 spectrofluorometer (Tokyo, Japan), with a programmable temperature controller attached. The protein solution was excited at 295 nm and the emission was recorded in the wavelength range of 300 nm to 500 nm. The PMT voltage was set at 500 V and the excitation and emission slits were set at 5 nm.

### 3.4. CD Spectroscopy

Far-UV CD measurements in the range of 200 nm to 250 nm were performed on the Jasco J-815 spectropolarimeter (Tokyo, Japan), with a cell of path length 0.2 cm.

### 3.5. Molecular Docking

Molecular docking calculations were achieved using Autodock vina. Ligand-free HSA (PDB id: 4K2C) was obtained from the protein data bank which was further optimized using a discovery studio visualizer and chain B was also deleted from the raw structure. Cuminol (PubChem CID: 325) and cuminaldehyde (PubChem CID: 326) were downloaded from the PubChem database (https://pubchem.ncbi.nlm.nih.gov/) on 27 October 2021. The PDBQT formats of the HSA and ligands were then obtained through AutoDockTools-1.5.6. Docking was performed by using a box size of 50 × 50 × 50 Å with grid center of X = 8.201, Y = −0.045, Z = 5.282. The value of exhaustiveness was kept at 100. From the various conformers obtained through docking, only the conformers with the least energy were chosen for further analyses.

## 4. Conclusions

The interaction of two cumin components with the important plasma protein, HSA, were observed using experimental and computational approaches. The motivation of the work was to see the interaction of two major cumin components (cuminaldehyde and cuminol) with HSA and to assess comparative interactions of aldehyde and alcohol from the same carbon skeleton. Both cuminaldehyde and cuminol interacted with HSA via a static quenching mechanism. However, while cuminaldehyde showed strong interaction with the protein, the interaction of the latter with cuminol was weak. Interaction was facilitated by the dominance of hydrophobic forces, as well as hydrogen bonding in the case of cuminaldehyde, whereas only hydrogen bonding was found in the case of cuminol. Both cuminaldehyde and cuminol partially unfold the secondary structure of HSA, although cuminaldehyde required very low concentrations as compared to cuminol, due to the stronger interactions of the former with HSA. Both cumin components bind at the same binding site inside HSA and most of the interacting amino acids were also common. Therefore, it can be concluded that aldehyde interacts strongly with HSA in comparison to the interaction of the same with alcohol.

## Figures and Tables

**Table 1:** Modified Stern-Volmer quenching constants and Lineweaver-Burk constants for the interaction of HSA with cuminaldehyde at various temperatures.

Temperature (°C)	Modified Stern-Volmer Quenching Constants		Lineweaver-Burk Constants	
	*K_Q_* (M^−1^)	*R* ^2^	*K_LB_* (M^−1^)	*R* ^2^
25	8.3 ± 0.04 × 10^3^	0.9969	8.3 ± 0.04 × 10^3^	0.9969
35	6.9 ± 0.02 × 10^3^	0.9804	7.0 ± 0.02 × 10^3^	0.9804
45	5.6 ± 0.02 × 10^3^	0.9985	5.5 ± 0.02 × 10^3^	0.9985

**Table 2:** Modified Stern-Volmer quenching constants and Lineweaver-Burk constants for the interaction of HSA with cuminol at various temperatures.

Temperature (°C)	Modified Stern-Volmer Quenching Constants		Lineweaver-Burk Constants	
	*K_Q_* (M^−1^)	*R* ^2^	*K_LB_* (M^−1^)	*R* ^2^
25	9.4 ± 0.42 × 10^2^	0.9848	9.4 ± 0.42 × 10^2^	0.9848
35	7.5 ± 0.38 × 10^2^	0.9868	7.6 ± 0.38 × 10^2^	0.9868
45	6.3 ± 0.29 × 10^2^	0.9905	6.3 ± 0.29 × 10^2^	0.9905

**Table 3:** Thermodynamic parameters of HSA–cuminaldehyde interaction.

*T* (K)	∆*G* (KJM^−1^)	∆*H* (KJM^−1^)	∆*S* (JM^−1^K^−1^)
298	−22.4 ± 0.03	−16.0 ± 0.10	21.6 ± 0.11
308	−22.6 ± 0.05		
318	−22.8 ± 0.01		

**Table 4:** Thermodynamic parameters of HSA–cuminol interaction.

*T* (K)	∆*G* (KJM^−1^)	∆*H* (KJM^−1^)	∆*S* (JM^−1^K^−1^)
298	−16.9 ± 0.02	−15.9 ± 0.04	3.3 ± 0.03
308	−16.9 ± 0.01		
318	−17.0 ± 0.01		

**Table 5:** Lineweaver-Burk constants for the interaction of HSA with cuminaldehyde/cuminol in the presence of site markers.

Site-Marker	Lineweaver-Burk Constants (M^−1^)	
	Cuminaldehyde	Cuminol
Warfarin	4.1 *±* 0.08 × 10^3^	2.3 *±* 0.06 × 10^2^
Ibuprofen	7.8 *±* 0.04 × 10^3^	6.0 *±* 0.07 × 10^2^

**Table 6:** The % α-helical contents of HSA (3 µM) in presence of various concentrations of cuminaldehyde/cuminol at 25 °C.

Concentration of Ligands (µM)	% α-Helical Contents	
	Cuminaldehyde	Cuminol
0	67.5 ± 0.11	67.5 ± 0.11
50	65.7 ± 0.19	66.6 ± 0.09
100	62.6 ± 0.17	64.9 ± 0.12
200	60.5 ± 0.21	63.7 ± 0.13
300	58.1 ± 0.10	62.7 ± 0.08
500	56.2 ± 0.12	61.6 ± 0.09
1000	-	59.5 ± 0.07
1500	-	56.9 ± 0.08
2000	-	54.0 ± 0.10

## Data Availability

Not applicable.

## Supplementary Materials

The following supporting information can be downloaded at: https://www.mdpi.com/article/10.3390/ijms23084078/s1. Figure S1: Stern-Volmer plots of HSA-cuminaldehyde systems at various temperatures. Figure S2: Stern-Volmer plots of HSA-cuminol systems at various temperatures. References [29,46] are cited in the Supplementary Materials.
