# Supplementary material for: Spectroscopic and Molecular Docking Investigation on the Interaction of Cumin Components with Plasma Protein: Assessment of the Comparative Interactions of Aldehyde and Alcohol with Human Serum Albumin
Source: Int J Mol Sci. 2022 Apr 7;23(8):4078. doi: 10.3390/ijms23084078 (PMC9028573; doi:10.3390/ijms23084078)
Supplement: Supplementary file 1 [file ijms-23-04078-s001.zip › ijms-1643695-supplementary.pdf]

Supporting Information

UV–Visible spectra were recorded between the wavelength range of 200 to 400 nm on Perkin-Elmer Lambda 45 Spectrophotometer equipped with autosampler and water-bath with temperature controller. Quartz cuvettes of 1 cm path length were used for the measurements.

Fluorescence measurements were performed on Hitachi spectrofluorometer (Model F 7000) equipped with a PC and programmable temperature controller. Unless stated, the fluorescence spectra were collected at 25 °C with a cell of path length 1 cm. The excitation and emission slits were set at 5 nm. Intrinsic fluorescence was measured by exciting HSA at 295 nm.

The circular dichroism studies of HSA in presence of cuminaldehyde/cuminol were carried out with JASCO J-815 spectropolarimeter equipped with a Peltier-type temperature controller. The instrument was calibrated with d-10-camphorsulfonic acid. All the CD spectra were collected in a cell of 2 mm path-length. The scan speed was 100 nm/min and response time of 1 s for all measurements. Each spectrum was the average of 3 scans.

The inner filter effect was corrected using the following equation [29]:

$$F_{corr} = F_{obs} \times 10^{(A_{exi}+A_{emi})/2} \quad \text{(S1)}$$

Where, F_{corr} and F_{obs} are the corrected and observed fluorescence emission intensities, respectively, A_{exi} and A_{emi} are the absorbance at the excitation and emission wavelengths, respectively.

Equation used to calculate thermodynamic parameters:

van 't Hoff equation is given as:

$$\ln K_b = \frac{-\Delta H}{RT} + \frac{\Delta S}{R} \tag{S2}$$

$$\Delta G = \Delta H - T\Delta S \tag{S3}$$

where ΔH is enthalpy change, ΔS is entropy change and ΔG is free energy change. R is gas constant and T is temperature in K.

The % α-helical content were calculated from the following equations:

$$\text{MRE} = \frac{\theta_{\text{obs}}(m\deg)}{10 \times n \times C \times l} \tag{S4}$$

where θ_{obs} is the observed ellipticity in millidegrees, C is the concentration of protein in molar, n is the number of amino acid residues and l is the length of the light path in cm. All spectra were smoothed by the Savitzky–Golay method with 5 convolution width. α-helical content was calculated from the MRE values at 222 nm using the following equation as described by Chen et al. [46]:

$$\%\alpha\text{-helix} = \left(\frac{\text{MRE}_{222\,\text{nm}} - 2{,}340}{30{,}300} \right) \times 100 \tag{S5}$$

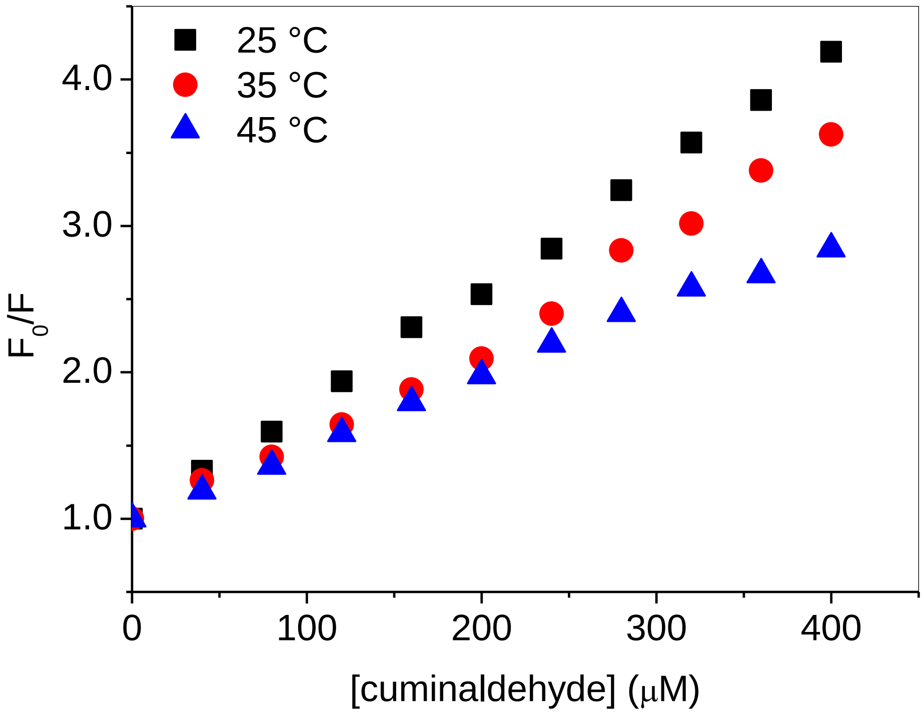

Figure S1. Stern-Volmer plots of HSA-cuminaldehyde systems at various temperatures.

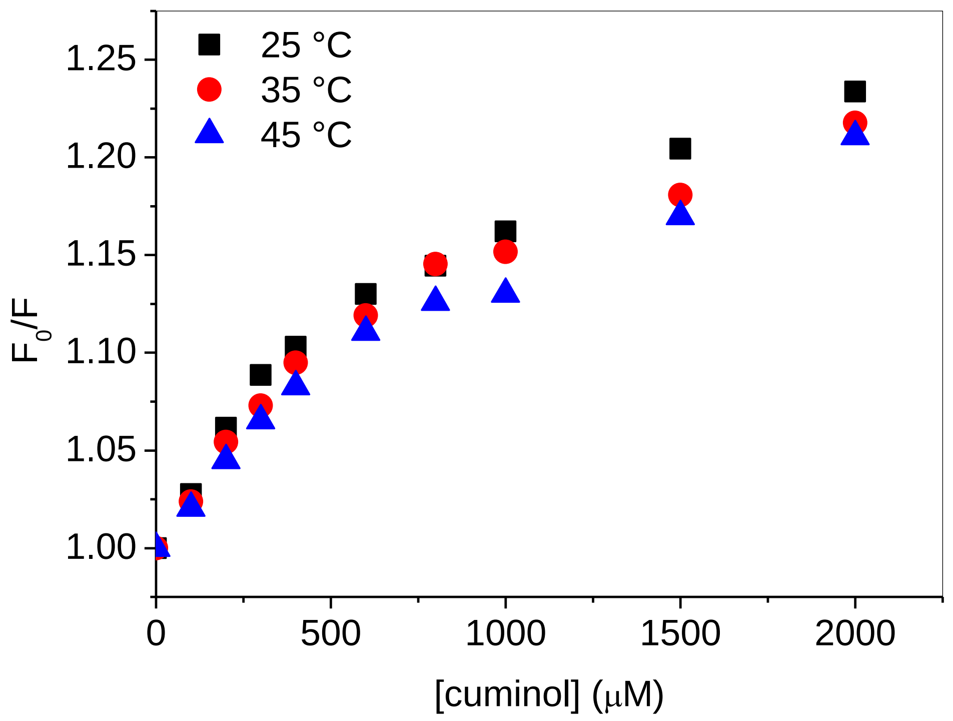

Figure S2. Stern-Volmer plots of HSA-cuminol systems at various temperatures.
